# ‘Getting shut down and shut out’: Exploring ACB patient perceptions on healthcare access at the physician-patient level in Canada

**DOI:** 10.1080/17482631.2022.2075531

**Published:** 2022-05-18

**Authors:** Tiyondah Fante-Coleman, Ciann L. Wilson, Ruth Cameron, Todd Coleman, Robb Travers

**Affiliations:** a Access and Equity Research Lab1, Department of Psychology2, Department of Health Sciences4 Wilfrid Laurier University, Waterloo, Ontario, Canada; b Dalla Lana School of Public Health5 University of Toronto, Toronto, Ontario, Canada; c Black Health Alliance, Toronto, Ontario, Canada; d Department of Psychology, Wilfrid Laurier University, Waterloo, Ontario, Canada; e AIDS Committee of Cambridge, Kitchener, Waterloo and Area, Kitchener, Ontario, Canada; f Department of Health Sciences, Wilfrid Laurier University, Waterloo, Ontario, Canada

**Keywords:** Health, African, Caribbean, Black, Healthcare System, Access, Community

## Abstract

**Purpose:**

The experiences of African, Caribbean and Black (ACB) Canadians are seldom explored in the Canadian context. Family physicians act as a gateway to the rest of the healthcare system and are necessary to provide proper patient care. However, Canada’s history with colonialism may impact the socio-cultural context in which patients receive care.

**Method:**

41 participants from Waterloo Region, Ontario, were engaged in eight focus groups to discuss their experiences in the healthcare system. Data were analysed following thematic analysis.

**Results:**

Style of care, racism and discrimination and a lack of cultural competence hindered access. oor Inadequate cultural competence was attributed to western and biomedical approaches, poor understanding of patients’ context, physicians failing to address specific health concerns, and racism and discrimination. Participants highlighted that the two facilitators to care were having an ACB family physician and fostering positive relationships with physicians.

**Conclusion:**

Participants predominantly expressed dissatisfaction in physicians’ approaches to care, which were compounded by experiences of racism and discrimination. Findings demonstrate how ACB patients are marginalized and excluded from the healthcare syste Iimplications for better access to care included utilizing community healthcare centres, increasing physicians’ capacity around culturally inclusive care, and increasing access to ACB physicians.

There are over 1.2 million African, Caribbean, and Black^1^ (ACB) people living in Canada, representing 3.5% of the population (Statistics Canada, 2019). ACB Canadians belong to diverse communities that have different relationships with Canada. Despite these differing relationships, they share a common experience of anti-Black racism. Racism has a tangible impact on the health and wellbeing of marginalized and racially classified communities. Research has shown that despite universal healthcare, health inequities exist for ACB Canadians (Lasser et al., 2006; McKeary & Newbold, 2010), likely due to Canada’s history of discriminating against ACB people. However, the healthcare experiences of ACB populations in Canada remain poorly documented in academic literature and require further exploration to reduce health inequity.

Racism in Canada can be traced back to British colonization and western medicine, which have set the stage for health inequities and poor access to healthcare (Byrd & Clayton, 2001). Today, ACB Canadians experience disproportionate adverse health outcomes, including increased reports of diabetes, hypertension, and high blood pressure (Toronto Public Health, 2013; Veenstra & Patterson, 2016). Moreover, the Covid-19 pandemic has highlighted the systemic consequences of anti-Black racism, as Black communities were excessively impacted (Cheung, 2020). Indeed, ACB communities experienced Covid-19 at a rate of 7,099 per 100,000, more than double the rate of white communities in the city (City of Toronto, 2021). Recent social and political movements have focused on the deleterious effects of racism on health and wellbeing. Indeed, advocates have deemed anti-Black racism to be a “public health crisis” (Boisvert, 2020), and there have been calls to “dismantle” anti-Black racism in medical care in Canada (Dryden & Nnorom, 2021).

Adequate access to healthcare, meaning access not hindered by a person’s race or socioeconomic status (SES), is necessary to reduce health inequities (Health Canada, 2001). Health equity is needed to address the unique needs of ACB Canadian communities who have been disproportionately impacted by anti-Black racism and poor access to care. Health equity occurs when all people can “reach their full health potential without disadvantage due to social position or other socially determined circumstance” (Ministry of Health and Long-Term Care Population and Public Health Division, 2018).

Healthcare providers are a crucial point of entry to the healthcare system since they deliver a critical service that cannot be achieved elsewhere and hold tremendous power over the health and wellbeing of their patients (Crooks et al., 2012; Martin, 2017). Access to primary care increases preventative care and screenings for cancer, ultimately improving patient outcomes (McIsaac, Fuller-Thomson & Talbot, 2001; Poole et al., 2010; Thanh & Rapoport, 2016). Healthcare providers also act as gatekeepers to specialist services, ensuring continuity of care and access to prevention efforts (Crooks et al., 2012; Nabalamba & Millar, 2000). However, ACB people’s relationship with their physicians can be negatively impacted by cultural differences (Jacobs et al., 2006), a lack of cultural competence (Cuevas et al., 2016), reliance on the biomedical model, and discrimination.

A lack of cultural competence hinders adequate access to care, particularly if providers’ ingrained biases remain unchecked (Cuevas et al., 2016; Van Ryn et al., 2011). Cultural competency aims to recognize the “sociopolitical contexts of poverty, racism, immigration, and culture” and the impact that they have had on access to healthcare and the utilization of services (Chin, 2000). Culturally competent care is essential at all points of delivery within the healthcare system (Cross, Bazron, Dennis & Isaacs, 1989, p. 8). However, the initial point of contact between patient and family physicians is especially significant because it sets the tone for the rest of the patient’s journey through the care continuum (Martin, 2017).

Healthcare typically centres on scientific knowledge, via the biomedical model, over a person’s experiential knowledge (Sword, 1999). The biomedical model relies upon simplistic explanations of biological pathology, which must be remedied by medical action (Engel, 1977). Prevention under this model also uses oversimplified ideations of “bad” and “good” behaviours. In the biomedical model, “bad” or unhealthy behaviours are a symptom of moral failure, and little attention is paid to their systemic causes. Thus, healthcare delivered under this model remains focused on individual behaviours (Sword, 1999). Evidence suggests that biomedical care remains a barrier for marginalized populations for many reasons, including distrust in how biomedical and Westernized care is delivered (Asgary & Segar, 2011). Distrust is impacted by a history of colonization in ACB people’s countries of origin (Durey & Thompson, 2012; Gamble, 1997). Marginalized populations increasingly want and need more culturally caring ways of receiving medical care (Durey & Thompson, 2012; Hyman, 2009), which can increase trust in the overall healthcare system.

Healthcare access can also be hindered by competing priorities and the models used by providers to administer care (Durey & Thompson, 2012). The traditional fee-for-service model inadvertently encourages shorter clinical interactions because physicians must bill for each service that they provide, emphasizing tests and shorter appointments (Linzer et al., 2015). Short clinical interactions also make physicians more likely to draw upon harmful stereotypes when administering care because physicians may rely on them to be more efficient (Chapman et al., 2013). Due to these constraints, it may be challenging to spend the time and resources necessary for providers to develop good relationships with their patients and address the complex challenges of their care (Linzer et al., 2015).

Experiences of discrimination within the healthcare system are well documented for African-Americans in the US. Mays et al. (2007) highlighted discrimination as a pernicious factor affecting African-Americans’ health status, findings that are supported theoretically by other public health researchers (Benjamins & Whitman, 2014; Sellers et al., 2003; Williams & Mohammed, 2009). For example, evidence suggests that African-Americans are far less likely to be believed and treated adequately for pain than other populations (Anderson et al., 2009). The failure to believe African-American patients may be attributed to physicians’ biases and their belief in myths concerning Black people’s tolerance of pain (Hoffman et al., 2016), impacting their care. Furthermore, beliefs that African-Americans were more likely to be drug-seeking affected the likelihood of physicians prescribing them pain medication (Todd et al., 2000). It is likely that discrimination also impacts access to care for ACB Canadians.

Despite their shared colonial history, there are differences between the experiences of African-Americans and ACB Canadians (Beiser, 2005; Hyman, 2009; McKeary & Newbold, 2010; Veenstra, 2009). So, the experiences of ACB Canadians accessing healthcare should be centred within the Canadian cultural context. Dryden and Nnorom (2021) have called for an increased focus on listening to the experiences of Black patients. This article responds directly to that call and explores patient perceptions in attempting to access care, problematizing the historical underpinnings of racism in the context of Canadian healthcare. To that aim, this article reports the findings of a qualitative research study that explored the experiences of ACB people attempting to access the healthcare system in Waterloo Region, Ontario. Research has emphasized that the experience of racism occurs at multiple levels of society, including at the micro, meso, and macro levels. In order to understand how racism operates, an analysis must consider how the experience of racism at the micro or individual level (e.g., between providers and patients) is tied to racism at the institutional or macro-level (Mahabir et al.). 2021; Paradies, 2006).

Thus, our research questions are 1) what are the barriers and facilitators ACB people face in accessing adequate healthcare at the point of physician-patient interaction? And 2) does race impact ACB people’s ability to access equitable care at the physician-patient level? We provide a historical overview of ACB Canadians’ interactions with the healthcare system and review the extant literature on access to primary care for this population in Canada. We will then share the results of a qualitative study that explore access to primary care physicians for ACB community members living in Waterloo, Ontario.

## Method

This article draws from data collected for the Adinkrahene: Improving Access to HIV Services for African, Caribbean and Black People in Waterloo Region study (The Adinkrahene Project). The Adinkrahene Project, a community-university partnership conducted with the AIDS Committee of Cambridge, Kitchener, Waterloo, and Area (ACCKWA) which aimed to explore access to healthcare services.

### Setting

Waterloo Region is situated 100 kilometres southwest of Toronto, Canada’s largest city and is home to an increasingly diverse population, with 25% of residents being foreign-born (University of Waterloo, 2016). Nearly 10,000 of those residents identify as African, Caribbean, or Black, an ethnically and culturally diverse population, who can trace their ancestry to the continent of Africa (Maticka-Tyndale et al., 2016). Waterloo Region has a thriving technology sector and two universities, attracting ACB residents to the region.

### Participants

Forty-one African, Caribbean, and Black participants ranging in age from 18 to 78 years (M = 36.50, SD = 14.43) participated in eight focus groups. Participants represented a socio-economically diverse sample, with incomes ranging from $0-9,999 to $70,000–99,000 CAD. Three participants declined to provide their incomes. Regarding city of residence, 27 participants lived in Waterloo, 13 in Kitchener, and 2 in Cambridge. Of the 41 participants, 24 self-identified as African, 16 as Caribbean, and two as Black-Canadian.

The African descendent diaspora in Canada is comprised of many people of different ethnicities and countries of origin (Fante-Coleman, Wilson, Marcotte, McKie & Travers, 2019; Maticka-Tyndale et al., 2016). Briefly, African-Canadian refers to those who have recently migrated from continental Africa in last few decades. Caribbean-Canadians have also recently immigrated to Canada and can tie their recent familial history to nations in the Caribbean, having arrived at those countries though the trans-Atlantic slave trade. Lastly, Black-Canadians have resided in Canada for hundreds of years. Many Black-Canadians’ familial history include the experiences of colonization and slavery, for instance, African Nova Scotians (Fante-Coleman et al., 2019).

### Researcher

An Afro-Caribbean graduate student was the primary researcher and coder of focus group data for this study. During the data collection and analysis phases, she lived in Waterloo Region and had spent a considerable amount of time in the area as both an undergraduate and graduate student. The primary researcher also worked closely with community organizations in Waterloo Region that focused on the health and wellbeing of Black communities. As a qualitative researcher, the primary author understands that human reality is socially constructed and that reality is often created (Berger & Luckmann,^1966^). The author also believes in multiple realities and ways of knowing. Communities have a knowledge and understanding of their realities and their impacts, particularly regarding their experiences in the healthcare system.

### Approach

A community-engaged research (CEnR) approach was used to inform the design and implementation of the Adinkrahene Project (Michener et al., 2012). CEnR centres on the role of community partnerships in the creation of knowledge. Research in a CEnR project emphasizes community empowerment (Michener et al., 2012). Ultimately, the goal of a CEnR project is to conduct useful and ethical research and ensure findings are owned and delivered back to the community (Michener et al., 2012).

Focus groups provided space to delve into the perceptions of ACB people accessing the healthcare system. Question guides for the focus groups were developed in partnership with ACCKWA. Semi-structured questions were utilized to understand participants’ perceptions of access to healthcare and their relationships with healthcare providers. Participants were asked questions such as: “How would you describe your relationship with your doctor? Are you satisfied?” and “Do you feel your race or culture has impacted the treatment you received from a healthcare professional?” Barriers and facilitators to accessing healthcare were also queried.

### Procedure

Participants were recruited through convenience sampling (Farrokhi & Mahmoudi-Hamidabad, 2012). Various outreach methods were used in recruitment, including recruitment posters, social media, and meetings with prominent ACB community and religious organizations. Before attending focus groups, all prospective participants were screened by phone to ensure they met the inclusion criteria (identified as ACB, lived in Waterloo Region, and were over 16 years of age). They were then assigned to a focus group based on their availability. Focus groups were held at the Centre for Community Learning and Action (CCRLA) on the Wilfrid Laurier University campus or at the Kitchener Public Library. Focus groups were conducted by either the study’s principal investigator (PI) or the primary author. One focus group was conducted by the PI and the executive director of ACCKWA. To ensure consistency, the first author shadowed the PI for the first two focus groups and led the other focus groups with the help of an undergraduate research assistant. All facilitators identified as ACB.

Prior to starting the focus groups, participants signed an informed consent form. Respondents also filled out a demographic survey, which took approximately 10 minutes to complete. Because of the sensitive nature of the project’s inquiry, all participants signed a confidentiality form before data collection. Unless declined, participants received $40 CAD in cash for remuneration. Focus groups were recorded and transcribed, lasting between 66 to 115 minutes. Notes detailed overall focus group topics and researcher impressions were recorded for each focus group. All study procedures were reviewed and approved by the Wilfrid Laurier University Research and Ethics Board.

### Analysis

Focus group data were transcribed by the primary author and transferred to Nvivo12. Participants’ were given pseudonyms to ensure confidentiality. Data were then analysed using thematic analysis (Braun & Clarke, 2006). A codebook was created to categorize and manage the data with the larger Adinkrahene Project research team. Codes were evaluated after each focus group and were discussed at length with the overall research team. Codes and themes were initially identified after the first three focus groups and were later refined after each additional focus group. Codes were chosen for their relevance to the research questions and the number of times participants discussed them. At a minimum, codes needed to emerge in five of the focus groups to be considered for inclusion. Researchers ensured that codes were accurate by sharing presentations provided via email for participants to review and provide feedback. The presentations highlighted emerging themes and at in-person member-checking sessions to ensure that they reflected participants’ experiences.

## Results

Participants described both barriers and facilitators to seeking care. Many of the themes focused on barriers to access related to interactions between healthcare personnel and patients. However, respondents also mentioned that interactions with physician-adjacent personnel, namely receptionists and nurses working in family physician practices, also impacted their access to care. Overall, participants noted a strong disconnect between the care they received and their ideal standard of care. Some participants’ dissatisfaction was due to concerns about how healthcare was delivered. However, many felt that their needs were not being met because physicians lacked knowledge about their culture, histories, and needs. Participants also experienced racism and discrimination from healthcare providers, which significantly impacted their experience accessing care.

**Figure 1.**
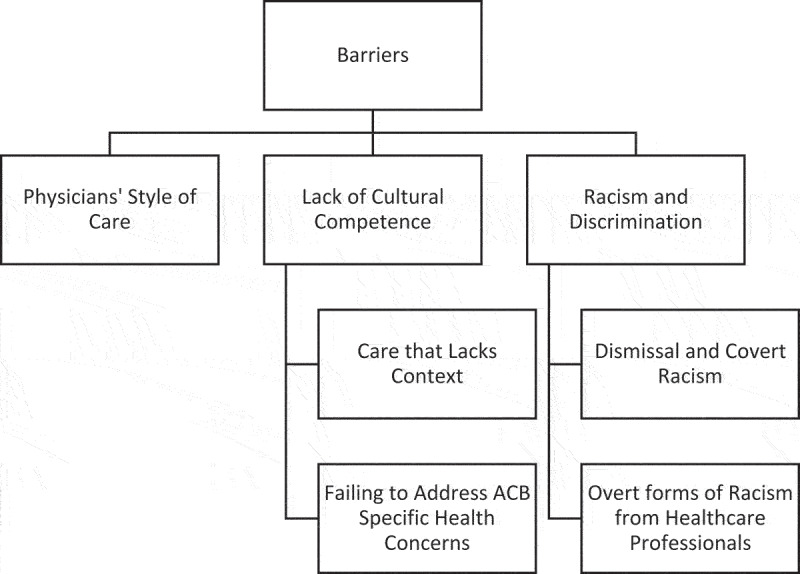
Barriers to care.

Data that spoke to barriers at the interpersonal level reflected the following three themes: *1) “Physicians” Style of Care,’ 2) “Lack of Culturally Humility in Care,”* and *3) “Racism and Discrimination.”* There were two subthemes within the theme *“Lack of Culturally Humility in Care”*: i*i) “Care That Lacks Context,”* and ii) *“Failing to Address ACB Specific Health Concerns.” “Racism and Discrimination”* also had two subthemes: i) *“Dismissal and Covert Racism”* and ii) *“Overt forms of Racism from Healthcare Professionals’ (*see, Figure 1.).

**Figure 2.**
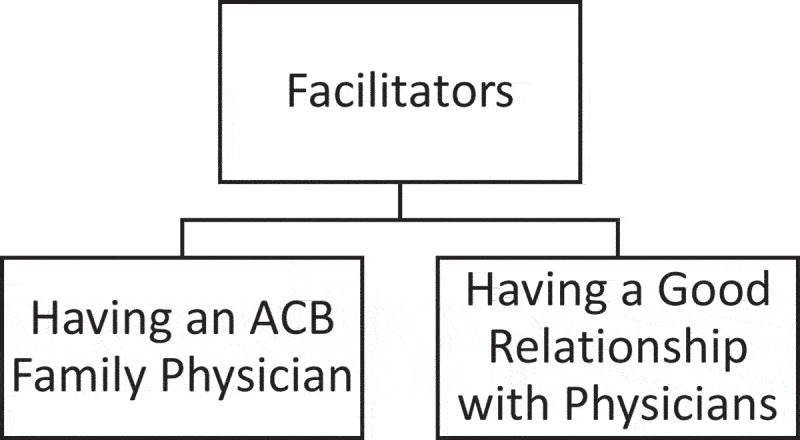
Facilitators to care.

Two facilitators were identified: *“Having an ACB Family Physician” and “Having a Good Relationship with Physicians.”* Figure 2. provides a graphical representation of the facilitators to care.

### Barriers to seeking care

Participants described numerous interpersonal barriers to seeking care. The most common barriers were physicians’ style of care, a lack of culturally competent care, and racism and discrimination from healthcare practitioners.

#### Physicians’ style of care

Many participants expressed displeasure at the clinical and biomedical approach physicians took. Respondents felt that physicians did not spend enough time with patients and that appointments were “short, authoritarian, and sometimes dismissive” [FG2]. Participants were particularly frustrated with the short, clinical nature of appointments because it compounded feelings of being unheard by medical professionals. As one participant summarized:

[Physicians] really don’t talk, you know, to make you feel comfortable and express exactly what you want. You just have to tell the most important [thing] about what you feel [because there is] not enough time [FG6].

Another said:

I think that they don’t spend enough time with patients. Once I took my son to the doctor, the doctor didn’t walk in the door. He just saw the rash on his face and just assumed that’s why we were there … She didn’t even sit down to hear us out [FG3].

In this case, the doctor had not yet examined the patient or asked their guardian preliminary questions about why they had scheduled an appointment, assuming they knew why the patient was there. Many participants noted that the short interactions made them hesitant to seek care. A participant in the fourth focus group described it this way:

Yeah, to go to the doctor. You have to kind of convince yourself to go. I try not to go unless I really should … [I have to] brace myself for having to deal with the stuff initially when you walk in and you’re talking to someone and you know they are not really listening to you [FG4].

Many participants felt that the imbalance in power between physicians and patients contributed to feeling unheard. Participants were also displeased at what they perceived to be the commodification of the physician-patient relationship, meaning a focus on the monetary value of appointments. Some participants hypothesized that this was the cause for shorter appointments. Another source of frustration was that appointments only addressed one concern at a time. Respondents felt that it was a significant demand on their time to book numerous appointments to have all their concerns heard. The same participant who had taken their son to see a physician above continues:

Yes, [the doctor] says, ‘I can’t discuss this right now. You need to get another appointment for that.’ So, you can’t discuss anything with the doctor. They just want you to get to the point, and before you know it, you’re out … Communication is really important, they don’t allow you to really explain how you feel … They are always, always in a hurry [FG4].

#### Lack of cultural competence

Related to the short, clinical nature of physician-patient interactions was the feeling that doctors lacked cultural awareness of the concerns of ACB people. Specifically, respondents disliked the Westernized focus of biomedical healthcare and felt that health concerns that were specific to ACB populations were overlooked. Furthermore, participants felt that physicians did not account for their lives’ contextual facets, which failed to address their concerns.

##### Care that lacks context

Respondents felt that the medical system and its delivery were not inclusive. Instead, they sensed that the advice given did not consider the cultural needs of patients:

I feel like they sometimes don’t understand us as Black people … Sometimes they have gone to school, and they’ve learned this … They don’t know exactly how each race, or each people are different from the other … They do not understand us as much as they should. And treat us the way that, at least have a different treatment than what everyone is having. That’s how I feel [FG5].

Participants sensed that physicians could rarely address the broader social causes and underlying causes of the illnesses that affected them, such as racism and generational trauma. Some participants felt that physicians, particularly White physicians, “don’t realize the stuff going on in our communities.” [FG4]. A participant from the fourth focus group explains:

Yeah, because you want to go and discuss stresses. You want to discuss family issues. You want to discuss historical stuff that made you stressed at some point. And you are talking to somebody who has not the slightest idea. Like it doesn’t matter how deep you go, they will never [understand] [FG4].

This participant lamented that physicians could not comprehend the perspective of their ACB patients. Other participants felt that medical training did not account for the differences among populations, believing that physicians were “poorly trained” in areas concerning ACB people and their cultural needs [FG4]. Another participant echoed that point, stating:

The problem is that a lot of these like Western-trained doctors and practitioners, they’re trained to individualize health. But when … you are talking about intergenerational trauma like in Black communities … if you don’t even know what that concept is, how are you going to [treat ACB patients? [FG4]?

The inability to relate to patients and their social world led to frustration and alienated respondents from the healthcare system. Participants worried that the focus on Eurocentric care presented another barrier to a system that is already difficult to access by ACB people:

I worry about already there’s so many like structural barriers to experiencing wellbeing right … I worry about people getting shut down and shut out of our system as it exists and not getting what they need to just like maintain a minimum standard of health … So healthcare providers who think that all the healthcare issues that do affect our communities are a result of our behaviour as opposed to all of the racism we’re experiencing [FG4].

##### Failing to address ACB-specific health concerns

Generally, participants felt that the way medical care was taught did not account for the health concerns that were specific to ACB people:

… Just basic knowledge around our communities, right? Understanding like why don’t [physicians] and nurses understand that things like breast cancer look different for Black women because of differences in certain genes. When it comes to breast cancer, you have to screen differently for Black women than you do for other women. Why don’t they know about sickle cell? And that people who carry the trait have medical issues as well as people who have the full-blown [sickle cell] disease? Why don’t they understand that heart attacks present differently in Black people [FG4]?

[ACB communities] also have some conditions that are specific to our communities. I think especially with women’s health. That’s something we need to be aware of, with anemia and different blood, things to do with our blood [FG8].

Other participants reiterated this concern and believed that physicians needed to be more knowledgeable about the needs of the ACB patients, stating:

‘Medical bodies need to do a better job … being educated about our health issues … They need to be aware’ [FG2].

Respondents were frustrated by the lack of attention paid to their community’s healthcare needs and felt that the system was purposely designed to marginalize ACB people. One participant pointed out that the lack of collection of race-based data concerning the health outcomes of ACB people was one way that the healthcare system failed ACB residents:

It literally sounds like sometimes the healthcare that is extended to Black people here is really similar to the healthcare that you would find in an undeveloped country. Because the things that you are talking about … are monitored in a country that doesn’t have the same [resources] [FG4].

The participant elaborated:

It gives you the feeling that things are happening deliberately … The idea that Black bodies should not be cared for. [We] do not feel pain … so many things that we don’t apparently feel … [We] never get the attention that is required [FG4].

Another participant in the same focus group lamented the lack of data concerning healthcare outcomes specific to the ACB population (also known as race-based data) and cited it as a barrier to receiving proper care, specifically concerning Black maternal health:

Something that I think about immediately when I think about Black women is because we don’t collect this kind of information in Canada, I worry about Black women and traumatic childbirth … I just know of so many stories, as well as my own, of there being far more distress and perhaps a full-blown medical emergency in childbirth because of not receiving the same care [FG4].

#### Racism and discrimination

Participants described instances of racism and discrimination that ranged from covert, in the form of dismissal, to occurrences of overt discrimination and stereotyping.

##### Dismissal and covert racism

Respondents also reported being frequently dismissed by physicians. Some interactions with physicians as dismissive and felt that there was “an assumption of ignorance on our part” [FG2]. Dismissal often occurred in instances where there was ambiguity. For instance, respondents often described being dismissed when they sought treatment for pain, which is self-reported and cannot be measured; this occurred even when the pain signalled a potentially life-threatening condition. Participants believed that physicians “discounted” their symptoms [FG5]. One participant reported that she had been dismissed by a nurse for pain in her leg when in actuality, she was suffering from a blood clot:

Can I just say, my leg is swollen, right? [The nurse] would think otherwise. She will start asking me, ‘did you hit yourself anywhere?’ But I didn’t tell you that I hit myself … my foot is swollen … I had a blood clot, it had split twice yet she kept on asking me stupid questions, ‘what kind of job do you do?’ I’m like ‘really? Why?’ [FG3].

Respondents were often accused of exaggerating or causing their pain. Some were accused of drug-seeking by the physicians treating them. One respondent recounted that a physician once asked if she used drugs when she was asking for pain relief:

Black people have to suffer. They have to live with pain unless and until it’s proven from an x-ray or from blood work, you won’t get painkillers. You will just go with Tylenol, Advil and that’s it. If you ask for anything stronger, [doctors] will just say, ‘do you use? You will be addicted’ [FG3].

Because even my mom, she’s like, she has chronic pain, she has like chronic diseases and stuff. [Every time] she goes to the doctor [they say] you’ll be fine, take Tylenol [FG7].

Respondents believed that physicians and healthcare providers needed to “see them suffer” [FG3] before they would take their pain seriously. Some participants attributed the dismissal of their pain as another way anti-blackness presented in the healthcare system. One participant from the third focus group explains:

They think for Black people, you cannot be seriously ill with what you are saying you are suffering from. It can’t happen that way. They have to think something is wrong with you. You get less care or attention because you are Black, and you have to be there to advocate for yourself [FG3].

##### Overt forms of racism from healthcare professionals

In addition to the previously described forms of covert racism, participants reported negative stereotyping from healthcare providers. These occurrences ranged from covert to highly overt. For example, a participant from the first focus group was told, “your people suck up the system and waste the taxpayers” money’ [FG1]. Though that was an obvious example of anti-Black racism, there were far more examples of stereotyping. One participant describes her experience:

I will say I think as far as the emergency room goes … they’re one of the worst places to go. I think they’re just overworked, and because of the amount of people that they see, they start to stereotype. Not even just like being Black or being on assistance or being a single parent … I [have] never had a good experience in the emergency room, because they look at me not as a person, but as a number or somebody they’ve had a bad experience in the past [FG1].

Other participants had numerous examples of stereotyping, ranging from physicians believing that babies born to African mothers in Canada had Ebola to participants being treated poorly due to their accent. Stereotyping was a frequent barrier to wanting to seek care:

It happens all the time, but I go to the doctor with my parents, they have a medical appointment and I go with them. And they’ve been in this country for 40 some years and … just because they talk with an accent. The doctor dismisses them like out of hand [FG7].

Most immigrants that I know don’t bother going to the doctor. Because you open your mouth, you are already branded. [Your] accent gives you away as somebody who is not really vested in the Canadian healthcare system, so it is … super, super difficult [FG4].

Beyond not wanting to seek care, stereotypes from medical professionals can have severe consequences for ACB people, especially if they lead healthcare providers to assume neglect or criminality.

I had a nurse call family and children’s services on me … She said that I was really needy, I was rude and annoying. [The report said] I was overly frantic like I was on drugs … I thought my child was dying on me … I don’t why she said any of that stuff because I just did … what the emergency worker told me to do … He’s only two years old, so he’s attached to heart monitors, so I’m traumatized by this, and I go home to find out that I get a report … I did everything in my power to do the right thing, and you hated me for no reason [FG1].

### Interpersonal facilitators to seeking care

Participants mentioned two factors that positively affected their ability to access care at the level of the physician-patient interaction. Having an ACB family physician and having a good relationship with their physician.

#### ACB family physicians

Having an ACB family physician was discussed numerous times across focus groups. Participants pointed to reasons such as shared lived experience, greater knowledge of their cultures, and intrinsic knowledge of ACB people’s bodies. Generally, participants felt that ACB physicians would be able to understand and empathize with their patients in a way that physicians who are not ACB are unable to.

Participants imagined what care for ACB people would be like with an ACB physician, asking “what would it mean to have access to either Black doctors or doctors who have an understanding of you know Black bodies and various Black cultures?” [FG4]. Other participants felt that they would be afforded greater attention from ACB family physicians than White physicians, feeling from past experiences that their interactions with White physicians have been “in general, more disrespectful” [FG2]. One participant who had an ACB family physician was pleased with the care she received:

I was just lucky to have a Black doctor twice … So when I go to my doctor, I can sit with my doctor, and he never rushes me. That is one of the [things] I like when I go there because he listens to you, he takes the time to understand your situation. And if my doctor doesn’t know what is wrong with me, he refers me, and he tries to eliminate anything that he can’t figure out himself [FG3].

Participants also felt that Eurocentric care, even from ACB physicians, was not what they believed constituted the best care:

I think my doctor is standard. She’s actually a Black doctor, but I think it’s good to see her because I’m sure, sadly, Black residents and doctors are underrepresented in the medical profession in Canada. But I think … she gives me conventional healthcare, so before I go to her, I will try to read up on whatever issue I have because I know her procedure will be the default one, and I don’t maybe always want to use it. So, I don’t think I am dissatisfied with it, but I also don’t think I am glowingly satisfied … but that’s mainly because it’s just the conventional, Western medical care [FG1].

#### A good relationship with physicians

Respondents felt that having a good relationship with their family physician was also an important factor in having good access to care:

I would much rather have a rapport with my doctor, like the doctor should be somebody that you walk in and you’re like okay, ‘I did this, I need you to tell me what’s going to happen to me.’ You should feel that comfortable with your doctor [FG1].

Other participants purposely attempt to cultivate a relationship with their physicians to ensure that they continue to receive the care that they were accustomed to:

A husband-and-wife practice team opened up near our home, and we got on that list as quickly as we could, and they have been our doctors for probably … 20 years, and so we definitely manage that relationship very carefully … We are very satisfied with the care, but we are also … not unaware of that this is not typical. So, we do maintain that relationship in many ways, we try to make contact with them socially as well [FG2].

Respondents felt that having a “good” relationship with their family physician consisted of having a rapport with physicians where they would offer treatment options and advice instead of an authoritative relationship where few options were offered to patients. Understandably, participants also wanted physicians who “didn’t see my skin color as a barrier, they didn’t see my race or my background as a barrier” [FG1]. Many participants said that they had better relationships with physicians who were younger for myriad reasons, including the type of advice they gave and the fact that younger physicians were more likely to ask for their input on the care they receive:

You know the younger doctors, she kinda still wanted me to have that right, which was nice because sometimes healthcare professionals go way over the top and they just act like they’re Jesus and ‘this is this, this is that, go you’re fine, get out’ [FG1].

## Discussion

This article explores the barriers and facilitators to accessing healthcare for ACB community members. In addition, we sought to explore the impact of racism on accessing care. Findings from eight focus groups elucidated the barriers and facilitators to accessing adequate healthcare at the interpersonal level. Focus group discussion revolved around four key themes related to barriers: physicians’ style of care, a lack of cultural competence, the dismissal of symptoms and concerns, and lastly, overt racism. For facilitators, participants mentioned that having a good relationship with their physician and having an ACB physician were facilitators to accessing healthcare that met their needs. These findings provide insight into the factors that participants believe hinder their access to care and why good physician-patient relationships are paramount to adequate care. Moreover, the results highlight how the experience of barriers to care due to racism are interrelated and amplify each other to affect patients’ perceptions of accessing care.

Participants often expressed dissatisfaction with the way physicians approached their medical care. Specifically, participants felt that appointments with their physicians were formal, uninviting, and short. There was a strong sense among participants that there was a power differential between physicians and patients, which compounded their feelings of not being heard. Because respondents anticipated their concerns would be overlooked, they often avoided seeking care.

These findings are consistent with those of other qualitative studies concerning African-Americans. Cuevas et al. (2016) and Asgary and Segar (2011) found that short clinical interactions and feeling unheard can lead to mistrust in patients, impacting treatment adherence and care-seeking behaviour. Given the established relationship between accessing physicians, preventative care, and improved health outcomes (Poole et al., 2010; Thanh & Rapoport, 2016), these findings are concerning.

Short appointments compounded feelings of miscommunication between patients and physicians. Brief clinical appointments that focused on one “issue” may be related to the model of healthcare their physicians used to administer care. For instance, the traditional fee-for-service model encourages billing OHIP for each patient’s interaction with their physician, inadvertently encouraging shorter and more frequent appointments (Linzer et al., 2015) related to neoliberalism and healthcare. Physicians are motivated by monetary gain over time spent with patients, likely due to the constraints placed on them by a lack of government funding (Whiteside, 2009).

The use of another model of healthcare, the comprehensive care model, was also identified as a barrier to access for ACB patients (Fante-Coleman, 2019). The author speculates that neither of these models may provide equitable access to care for ACB people. This is of concern because they are the most commonly used in Ontario (Hutchison & Glazier, 2013). Shorter clinical appointments may cause physicians to rely on stereotypes, impacting their practice and increasing the likelihood that ACB people will receive poorer care (Beagan & Kumas-Tan, 2009; Cuevas et al., 2016).

A lack of cultural competence was a frequent complaint of participants, who identified two key aspects of concern: care that did not address the context of participants’ lives and a dearth of attention on the health issues that particularly impacted ACB people. Participants described a lack of inclusiveness in the healthcare system as a barrier to care. Physicians’ care seemed incomplete because it addressed their physical ailments but not the structural factors that impacted respondents’ health (Beagan & Kumas-Tan, 2009). Physicians were often unaware of the socio-political context of participants’ lives. Furthermore, this style of care reinforced a simplistic and individualized conception of health, which was often incongruent with participants’ worldviews. These findings highlight similarities with extant research conducted in Toronto, suggesting that the implementation of healthcare has similar effects regardless of location (Women’s Health in Women’s Hands, 2003).

In recent years, cultural competency has made way for *cultural humility,*which posits that providers must “… continuously engage in self-reflection and self-critique as lifelong learners and reflective practitioners” (Tervalon & Murray-Garcia, 1998). Furthermore, cultural humility requires that providers examine the power imbalances present in provider-patient relationships and position themselves as learners of their patients’ cultural beliefs and values rather than western scientific knowledge alone (Juarez et al., 2006).

Respondents felt that physicians lacked “basic knowledge” of the illnesses that most impacted the ACB community. Overall, there was a general feeling that physicians were unaware of the specific health needs of ACB people. The dearth of race-based data specific to ACB people may contribute to the failure of physicians to address their health priorities(Nestel, 2012). This contributed to further distrust of the medical system in participants (Cuevas et al., 2016). Because of the lack of focus on race and the specific health concerns of ACB people, some participants expressed concern that their inability to receive adequate care was a deliberate oversight, affirming their distrust in the system.

Rodney and Copeland (2009) assert that the lack of race-based data in Canadian healthcare is emblematic of how Canada utilizes race and “multiculturalism” to market its tolerance while simultaneously obscuring the health inequities present in racialized Canadians. In the US, national health agencies utilize disaggregated data to identifyand address inequities. For instance, the Centers for Disease Control and Prevention monitor and track the maternal health of African-Americans to identify health inequalities (Centre for Reproductive Rights, 2014). However, no system in Canada currently uses race-based data to reduce disparities.

Previous research has found that Canadians have mixed opinions on collecting race-based data (Kirst et al., 2013). Both white and racialized participants were unsure of the necessity of collecting race-based data to identify health inequities and had privacy concerns. The present study did not ask participants their opinions on collecting race-based data, but participants cited the lack of it as a barrier to accessing equitable care. The failure to explore the specific health needs of ACB people is a reproduction of structural inequalities. The lack of applicable knowledge related to the health disparities ACB communities experience is relevant to inherent racism within the healthcare system (Czyzewski, 2011). The purposeful lack of data concerning ACB people in the present could also reflect colonialism’s presence in the Canadian healthcare system. In the past, medical professionals did not hesitate to use ACB people to create knowledge that would benefit the White population (Gamble, 1997). In the present, knowledge is necessary to create meaningful action towards health equity (Rodney & Copeland, 2009).

The intentional aggregation of health data allows the government and its associated ministries to absolve themselves from taking the requisite steps to improve health outcomes. Reliance on aggregated data that focuses on all racialized Canadians is insufficient and makes it challenging to identify inequities and target interventions to promote specific improvement in healthcare outcomes (Rodney & Copeland, 2009). Race-based data is imperative to combat health inequities.

Dismissal was a reoccurring theme in focus groups; respondents believed that physicians, particularly non-Black physicians, often devalued or dismissed their symptoms and concerns. These findings are similar to the experiences of African-Americans, for whom dismissal has been identified as a barrier to healthcare (Anderson et al., 2009). Similarly, participants reported perceptions that physicians thought they were over-reacting or exaggerating their pain (Anderson et al., 2009; Hoffman et al., 2016)

Pain is subjective, and there is ambiguity in determining just how much pain a person experiences at any given moment. This ambiguity allows racism to work in ways that make it difficult to detect. Racism, even if not expressed explicitly, impacts the care ACB people receive in the Canadian healthcare system (Penner et al., 2009). The impact of provider bias is significant because it is likely that most ACB patients have “racially discordant” (where patients and providers are different races) relationships with providers (Penner et al., 2009). Similar biases have been documented in physicians who treat African-American patients (Cuevas et al., 2016; Penner et al., 2009).

ACB people are acutely aware of instances of discrimination and are more likely to perceive discrimination when it occurs (Williams & Mohammed, 2008). Regardless of intent and its presentation (implicit or explicit), the effects of racism do not differ for patients. Implicit racism still has tangible impacts on the health of ACB people and their ability to adequately access care, as evidenced by participants’ experiences in this study.

Research has shown an association between healthcare utilization and perceived discrimination (Lee et al., 2009). In healthcare settings, participants were confronted with stereotypes from their family physicians and emergency department personnel in explicit instances of racial discrimination. Furthermore, negative experiences with healthcare professionals overlapped with other social systems, such as child protective services. Most patients referred to child protective services in the US were African-American (Fortin et al., 2016). Physicians are mandatory reporters, meaning they must report suspected instances of abuse. However, physicians who hold racist biases can wield their institutional powers to decide who is “guilty” of abuse.

Similarly, in Canada, ACB youth are overrepresented in child protective services (Ontario Human Rights Commission, 2018). These systems overlap and reinforce their inherent racism to marginalize further and oppress ACB people. This has implications for healthcare access because the fear of being reported to child protective services may prevent parents who are already wary of medical institutions from bringing their children to hospitals.

The relationship between physicians and patients is the foundation of good family physician practice because they serve as the connecting point to the rest of the healthcare system (Martin, 2017). Participants were cognizant of this and wanted to ensure that they received the best care possible. Many respondents described going to great lengths to maintain their relationships with their family physicians because they believed those relationships impacted their care. Including patients’ perspectives in the care process is highly correlated with better healthcare outcomes, and patients who share control of their care are more likely to adhere to treatment (Cuevas et al., 2016). Thus, physicians should consider an egalitarian approach to delivering in collaboration with their patients.

Participants felt that having an ACB family physician would eliminate some of the barriers they faced in accessing care. Respondents believed that the shared lived experience of racism between ACB physicians and patients would lead to an inherent understanding of patients’ perspectives (Malat & van Ryn, 2005). These results were similar to a study that showed African-Americans preferred same-race physicians, likely due to the colonial history of healthcare in both the US and Canada (Malat & van Ryn, 2005). Participants who did have ACB family physicians described being generally more satisfied with their care. Evidence corroborates that African-American patients receive better care when receiving care from racially-concordant physicians (Jacobs et al., 2006).

In Ontario, there has been a recent focus on the dearth of ACB physiciansand the lack of representation in medical schools. A concerted effort from Black physicians and medical schools has led to an increase in admissions for ACB medical students, which represents significant possibilities for greater healthcare access for ACB patients in Ontario. More ACB physicians are needed to increase equitable access to care for this community (Walji, 2015).

Despite these strides, there is a possibility that having an ACB family physician may not improve access to equitable care if the care provided remains unchanged. In focus groups, participants clearly stated that they wanted more culturally competent care. However, participants have not been queried on what competent care meant to *them*. Future research should explore the ways ACB people conceptualize competent care, with the intent of designing strategies for practice.

The race of their physician did not always matter to participants, who expressed they had good relationships with physicians who saw beyond their race and could communicate with them effectively, which are components of culturally competent care (Jacobs et al., 2006). Culturally competent care has been described as care that values diversity, can perform accurate cultural assessments, is conscious of power dynamics, encompasses cultural knowledge and has adapted to the needs of diverse patients. Culturally competent care has been proposedas a solution tothe healthcare inequities that ACB people face in accessing the healthcare system. Thus, clinicians interested in improving the healthcare outcomes of their ACB patients, as all physicians should, would be remiss to continue providing care that is strictly biomedical and colour-blind. Care that focuses on cultural humility, where physicians reflect on their social location and power and centre learning about the cultures of their patients (Juarez et al., 2006) has the potential to further increase equitable access to care.

### Implications

This article contributes to the small amount of extant literature on ACB people and access to healthcare in Canada. It aimed to elucidate the barriers and facilitators at the interpersonal level, examining the relationship between physicians and patients. The results of this study are significant because they highlight how ACB people are marginalized and excluded from systemic structures. These findings have important implications for healthcare practitioners and medical policymakers.

The findings of this study demonstrate that ACB patients feel that the care they receive from physicians is lacking. ACB patients perceive their inability to receive care as deliberate and are wary after centuries of medical mistreatment, and physicians must work hard to regain their trust. Physicians must acknowledge that barriers to access exist and work to eradicate them. As well, physicians should adopt a form of culturally competent care into their practice. The aspects of healthcare that are taken for granted, such as long wait times, short appointments, and lack of cultural consideration, should be examined critically. Physicians should consider providing patients with options for longer appointments to build trust and rapport. In these sessions, the focus should be on a style of practice that centres on collaboration to address the patient’s concerns. Of course, this will need broader support from policymakers and should be incorporated into medical training. Physicians can also improve their cultural competence by understanding the impact of racism on ACB communities, learning what illnesses are of concern to them, comprehending how ACB communities conceptualize health and wellbeing beyond the traditional biomedical model and lastly, work to eliminate racism and stereotypes from their practice.

The financial model physicians use may also pose a challenge to healthcare access for Black communities. A potential solution was the model utilized by community healthcare centres, which is more interdisciplinary and uses a holistic approach to health by employing allied health professionals and physicians and nurses (Khandor et al., 2011). Participants were not queried on their preferred model of care or their experiences with certain models of care in this study, which should be a topic of further inquiry. As well, this study highlighted a stated need for disaggregated race-based data and more ACB physicians to address= health inequities and the specific needs of this population. Lastly, participants noted their desire for more ACB physicians who can provide care. Thus, stronger measures should be taken to increase the enrolment of prospective Black physicians to meet patient demand.

### Limitations

This investigation draws on data collected for the Adinkrahene Project. The study’s focus on health may have affected the engagement of potential participants due to distrust (Gamble, 1997). Furthermore, this project was conducted in partnership with a local community organization, the AIDS Committee of Kitchener, Cambridge, Waterloo and Area. This partnership may have further hindered participation because of stigma concerning HIV/AIDS (Baidoobonso et al., 2013).

Because of time constraints, focus groups were conducted with men and women-identified participants, who varied greatly in age. Some participants were more outspoken than others, and certain viewpoints may have dominated discussions (Smithson, 2000). There may have been implied consensus among participants in discussions where there was none. Facilitation skills, such as redirecting, were employed to encourage equal participation (Smithson, 2000).

Participants’ familiarity with each other may have also hindered participation. Despite widespread recruitment, many participants who were recruited separately knew other participants in the same focus groups; this is most likely due to how tight-knit the ACB community is in Waterloo Region, where many people who are active in this community frequent the same social circles. Many candid discussions took place during focus groups, suggesting that participants were comfortable sharing. Participants may have been hesitant to disclose information and feelings about certain topics due to concerns about confidentiality. Participants were asked to sign confidentiality forms that highlighted the importance of not sharing personal information to minimize these challenges.

Lastly, because of the community-engaged nature of this work, many participants were highly engaged members of their community who wanted to see improvements in access to healthcare. Thus, participants’ views may not represent those held by all ACB people living in Waterloo Region.

### Future Directions

The findings of this study highlighted questions that should be addressed in future research. Participants were not queried on their preferred model of care or their experiences with certain fee models. This article suggests that the type of model utilized by physicians has implications for access and so this should be explored further. Though concerns were raised about the lack of race-based data on health outcomes for ACB people, participants were not directly asked about their opinions on the collection of race-based data, which has been received with ambivalence in other populations et al., 2013). Future research should focus specifically on the perceptions of ACB people in collecting race-based data to monitor health inequities in the Canadian context.

In the focus groups, participants clearly stated that they wanted more culturally competent care. However, participants have not been queried on what competent care was to *them*. Future research should explore the ways ACB people conceptualize competent care, with the intent of designing strategies for practice. Future research should also focus on the perceptions of ACB people in collecting data in the Canadian context.

## Conclusion

Racism plays a role in how ACB folks experience care in healthcare settings in Ontario. This study reveals that ACB people feel that they are receiving inadequate healthcare due to short clinical appointments, lack of cultural competence, and experiences of discrimination ranging from dismissal to overt racism. Moreover, this study provides evidence that Black community members are experiencing racism and discrimination from the professionals tasked with providingnecessary healthcare services. A lack of adequate access to care can have significant implications on the healthcare outcomes of ACB people in Canada. Thus, more research is needed to understand how to address these barriers to access and eliminate racism and discrimination from the healthcare process.

Culturally competent care, race-based data collection, different models of service provision and increased access to ACB health providers are some solutions that may reduce the disparities to access that currently exist. The findings of this research study can be used to inform the literature on ACB people and access to healthcare, ways to reduce racism in the Canadian healthcare system, and demonstrate the need for more ACB physicians and other physicians of colour. It is also clear that the way medicine is taught in schools in Canada needs to have a stronger focus on the impacts of racism on medical care. Further research is needed to determine the best service provision models to reduce access disparities and understand what physicians can do to encourage better relationships with ACB communities.

## Note

1. Individuals of African descent who can tie their presence in Canada to the trans-Atlantic slave trade that existed in both Canada and the USA (Maticka-Tyndale et al., 2016)
